# Prevalence, enumeration, and pheno- and genotypic characteristics of *Listeria monocytogenes* isolated from raw foods in South China

**DOI:** 10.3389/fmicb.2015.01026

**Published:** 2015-09-29

**Authors:** Moutong Chen, Qingping Wu, Jumei Zhang, Shi Wu, Weipeng Guo

**Affiliations:** ^1^Guangdong Institute of Microbiology, State Key Laboratory of Applied Microbiology Southern China, Guangdong Provincial Key Laboratory of Microbial Culture Collection and Application, Guangdong Open Laboratory of Applied MicrobiologyGuangzhou, China; ^2^School of Bioscience and Bioengineering, South China University of TechnologyGuangzhou, China

**Keywords:** *Listeria monocytogenes*, most probable number, enterobacterial repetitive intergenic consensus PCR, random amplified polymorphic DNA, antimicrobial susceptibility profile

## Abstract

*Listeria monocytogenes* is an important foodborne pathogen that can cause serious illness in immunocompromised individuals, pregnant women, the elderly, and newborns. The aim of this study was to: (i) evaluate the prevalence and contamination level [most probable number (MPN)] of *L. monocytogenes* in 567 retail raw foods (fishery products, *n* = 154; raw/fresh meat, *n* = 123; frozen foods, *n* = 110; edible fungi, *n* = 108; vegetables, *n* = 72) collected from South China and (ii) to gain further knowledge on the phenotype and genotype distributions of this important foodborne pathogen. Approximately 22% of the samples were positive for *L. monocytogenes*. The contamination levels were between 0.3 and 10 MPN/g in 75.0%, between 10 and 100 MPN/g in 11.0% and less than 100 MPN/g in 14.0% of the countable samples. Five serogroups were identified among the 177 foodborne *L. monocytogenes* isolates, with 1/2a-3a (42.4%) and 1/2b-3b (26.0%) serogroups being the most dominant. Serogroups I.1 and II.2 were only found in the edible mushrooms, while serogroup III was dominant in the fishery products, suggesting that specific serogroups of *L. monocytogenes* may have distinct ecological niches. Ten (5.6%) *L. monocytogenes* isolates exhibited multidrug resistance. Genetic relatedness analysis revealed the absence of distinct associations between specific food types, antibiotic resistance, serogroups, and genetic diversity. The present study provided the first baseline data on the prevalence, contamination level, and characteristics of *L. monocytogenes* isolated from raw foods in South China. Some multidrug resistant strains belonged to the epidemiologically important serogroups (I.1 and II.1), implying a potential public health risk. In addition, these findings also provide basic information for the Chinese food safety associated authorities to draft appropriate standards to control *L. monocytogenes* contamination and improve microbiological safety of raw foods.

## Introduction

*Listeria monocytogenes*, a facultative intracellular foodborne pathogen, is capable of causing serious disease in humans, especially in immunocompromised individuals, pregnant women, the elderly, and newborns. It has the ability to inhabit a wide range of environments and is commonly found in food, nature, and the food processing environment (Abadias et al., 2008). Listeriosis is a severe invasive disease and the manifestations include neurological infections like encephalitis, meningitis, septicemia, and abortion, with a mortality rate of up to 20–30% (Vazquez-Boland et al., 2001; Kang et al., 2013).

Foodborne outbreaks of listeriosis have been documented in Asia, Europe, and the USA (Makino et al., 2005; de Castro et al., 2012; Lomonaco et al., 2013). In recent years, several sporadic cases of listeriosis have been reported in China (Wu et al., 2008; Hsieh et al., 2009; Feng et al., 2011). *L. monocytogenes* isolates associated with outbreaks or sporadic cases of listeriosis have been detected in different kinds of foods including dairy products, raw meat, vegetables, and fishery products (Schlech, 2000; Bell and Kyriakides, 2005; Yücel et al., 2005). Identifying the source of infection is essential to undertake preventive measures and control the exposure to such infection (Franciosa et al., 1998). Thus, determining the prevalence and contamination level of *L. monocytogenes* in retail foods are of utmost importance to control and track the definite source of *L. monocytogenes*. In China, previous studies have focused on the prevalence of *L. monocytogenes* in retail foods. However, these studies only focused their investigation to limited regions, such as Heilongjiang Province, Guangzhou City, and Gansu Province (Hu et al., 2013; Shi et al., 2013; Wang et al., 2013). In this context, it is notable that South China is located in a subtropical region where the climate is suitable for *L. monocytogenes* growth. Several studies have focused on the occurrence of *L. monocytogenes* in ready-to-eat foods (Huang et al., 2005; Mei et al., 2006; Lianou and Sofos, 2007; Jin et al., 2009; Sauders et al., 2009). Additionally, numerous previous studies have reported the contamination level of *L. monocytogenes* in ready-to-eat products in other countries (Lianou and Sofos, 2007). Wang et al. (2013) reported occurrence and counts of *L. monocytogenes* in retail raw foods in Heilongjiang Province (Northeast of China) from 2008 to 2009. However, there are limited studies that focused on the contamination level of *L. monocytogenes* in retail raw foods in South China, especially the contamination level in edible fungi and vegetables, thus, hampering the potential risk analysis of *L. monocytogenes* in retail raw foods. It is necessary to monitor the occurrence of *L. monocytogenes* in retail raw foods because of possible chances of cross-contamination during food processing and food storage.

Since the first multiresistant *L. monocytogenes* strain was isolated from a patient with meningoencephalitis in France in 1988 (Poyart-Salmeron et al., 1990), antimicrobial resistant strains have been commonly recovered from food, natural environment, and clinical cases of listeriosis (Rodas-Suárez et al., 2006; Zhang et al., 2007; Conter et al., 2009; Morvan et al., 2010; Granier et al., 2011). The resistance profiles are varied and may be influence by overuse of antimicrobials in humans and livestock animals, as well as by geographical differences. In China, the rates of antibiotic resistance in 467 foodborne *L. monocytogenes* isolates were 4.5% in 2005, and the antimicrobial resistance was most frequently observed for ciprofloxacin and tetracycline (Yang et al., 2008); similar results were also reported by Yan et al. (2010). Zhao et al. (2012) reported that the rates of antibiotic resistance in 1069 foodborne *L. monocytogenes* isolates were 6.92% in China. The resistance was most prevalently observed for antibiotics such as tetracycline, doxycycline, erythromycin, chloramphenicol, and ciprofloxacin. Based on this trend of antibiotic resistance, it is advisable to monitor the patterns of antibiotic resistance of *L. monocytogenes* in food sources from different regions in China.

*Listeria monocytogenes* has 13 serotypes that can be divided into five serogroups, such as I.1 (1/2a-3a), I.2 (1/2c-3c), II.1 (4b-4d-4e), II.2 (1/2b-3b-7), and III (4a-4c; Doumith et al., 2004). As 95% of the strains responsible for human listeriosis and found in food samples belong to serotype 1/2a, 1/2b, 1/2c, and 4b (Pontello et al., 2012; Althaus et al., 2014), serotype analysis is not very efficient method to differentiate *L. monocytogenes* strains isolated from foods and clinical samples. Recently, numerous molecular subtyping techniques have been developed for the surveillance or tracing the sources of *L. monocytogenes*; these include pulsed field gel electrophoresis (PFGE; Gerner-Smidt et al., 2006), amplified fragment length polymorphism (AFLP; Parisi et al., 2010), random amplified polymorphic DNA (RAPD; Farber and Addison, 1994), multi-locus sequence typing (MLST; Parisi et al., 2010), and enterobacterial repetitive intergenic consensus polymerase chain reaction (ERIC-PCR; Chen et al., 2010). Of these techniques, ERIC-PCR is a relatively simple, highly reliable, and cost-effective method that has been demonstrated to generate distinct DNA fingerprints within a single bacterial species (Jersek et al., 1999).

The objective of the present study was to: (i) determine the prevalence and contamination level of *L. monocytogenes* in retail raw foods in South China and (ii) to determine the genetic variation and phenotypic characteristics of *L. monocytogenes* isolates.

## Materials and Methods

### Sampling Procedure

From July 2011 to August 2012, a total of 567 retail raw food samples were collected from rural markets, open-air markets, and large supermarkets in South China, including five districts of Guangzhou City and 11 cities in Guangdong, Fujian, Guangxi, and Hainan Province. Samples comprised of fishery products (*n* = 154, including prawn, Grass carp, fresh squid, speckled fish, yellow croaker, catfish, oyster, *Tilapia mossambica*), raw/fresh meat (*n* = 123, including fresh pork, fresh beef, chicken, duck, Minced pork, fresh pork ribs, fresh pork ball, mutton), frozen foods (*n* = 110, including Chinese dumpling, frozen mutton, frozen chicken wing, frozen han sausage, frozen drumstick, wonton, frozen salted pork), fresh edible fungi (*n* = 108, including *Flammulina velutipes, Pleurotus eryngii, Hypsizygus marmoreus, Coriandrum sativum, Lentinus edodes*, oyster mushroom, *Pleurotus geesteranus*), and vegetables (*n* = 72, including Lettuce, cucumber, coriander). Samples were stored in insulated shipping coolers containing frozen gel packs placed on the sides, middle, and the tops of the samples. All the samples were kept below 4°C during transportation and test was initiated within 4 h after receipt.

### Qualitative and Quantitative Analysis for Food Samples

An enrichment method according to the National Food Safety Standard of China – Food microbiological examination: *L. monocytogenes* (GB 4789.30-2010; National Standard of the People’s Republic of China, 2010) with minor modifications was used for qualitative detection. In brief, samples were analyzed for the presence of *L. monocytogenes* by homogenizing 25 g samples in 225 mL *Listeria* enrichment broth I (LB1; Huankai, Co. Ltd., China). Homogenates were incubated at 30°C for 24 h. Thereafter, 0.1 mL LB1 enrichment culture was transferred to 10 mL *Listeria* enrichment broth II (LB2) at 30°C for 24 h. A loopful of the LB2 enrichment culture was streaked onto Chromagar^®^*Listeria* selective agar plates (CHROM-agar, Paris, France) and incubated at 37°C for 48 h. Three to five presumptive colonies that were typically blue in color with a white halo were selected for identification of *L. monocytogenes* by Microgen ID *Listeria* identification system (Microgen, Camberley, UK) according to the manufacturer’s instructions.

To determine the most probable number (MPN), the method was adapted from a previous study conducted by Gombas et al. (2003). Briefly, a nine-tube MPN method was used. The nine tubes were divided into three sets of three tubes each. The second and third sets of tubes contained 10 ml of Fraser broth medium. Three aliquots (10, 1, and 0.1 mL) of the sample homogenate were dispensed into three sets, representing 1.0, 0.1, and 0.01 g of the original sample, respectively. The tubes were incubated at 30 ± 2°C for 24 ± 2 h, and subsequently 0.1 mL of content from each tube was transferred to a new tube containing 10 ml of fresh Fraser broth. The tubes were incubated at 30 ± 2°C for 26 ± 2 h. Darkened Fraser tubes were subjected to confirmation. If a Fraser broth failed to darken, it was examined again after an additional 26 ± 2 h of incubation. The MPN values were determined based on the number of positive tube(s) in each of the three sets and the MPN table (U. S. Department of Agriculture, 1998; Hitchins, 1998).

### Serogroup Analysis using Multiplex PCR

Genomic DNA was extracted from *L. monocytogenes* using Bacterial Genomic DNA Purification Kit (Dongsheng Biotech. Inc., Guangzhou, China) according to the manufacturer’s instruction. DNA concentration was determined at O.D. 260 nm using Nano Drop^®^ND-1000UVeVis Spectrophotometer (Thermo Fisher Scientific, Inc., Waltham, MA, USA). Serogroup analysis of 177 isolates was performed using a multiplex PCR as previously described by Doumith et al. (2004),13 serotypes of *L. monocytogenes* were categorized into five distinct phylogenetic groups, viz. I.1 (1/2a-3a), I.2 (1/2c-3c), II.1 (4b-4d-4e), II.2 (1/2b-3b-7), and III (4a-4c). The PCR mixture (50-μL) contained 1.5 unit GoTaq^®^Hotstart polymerase (Promega, Madison, WI, USA), 1 μM for *lmo0737, ORF2819*, and *ORF2110*; 1.5 μM for *lmo1118*; and 0.2 μM for *prs*, 2.5 mM MgCl2, 0.2 mM each dNTP, and 40 ng of template genomic DNA. PCR was performed with the following thermal cycle: initial denaturation step at 94°C for 3 min; 35 cycles of 94°C for 35 s, 53°C for 50 s, and 72°C for 60 s; and a final cycle of 72°C for 7 min in a thermocycler (Applied Biosystems, Foster City, CA, USA). Five microliters of the reaction mixture was mixed with 5 μL of loading buffer and separated on a 2% agarose gel in TAE buffer. The PCR product was visualized by Goldview^®^staining (0.005%, v/v). The primers are shown in Supplementary Table S1.

### Enterobacterial Repetitive Intergenic Consensus Sequence Polymerase Chain Reaction (ERIC-PCR)

Enterobacterial repetitive intergenic consensus sequence polymerase chain reaction (ERIC-PCR) typing was carried out on the *L. monocytogenes* isolates and five reference strains using the protocol described by Chen et al. (2014). The ERIC primers was described by Versalovic et al. (1991), ERIC1R: 5′-ATGTAAGCTCCTGGGGATTCAC-3′, and ERIC2: 5′-AAGTAAGTGACTGGGGTGAGCG-3′. The PCR mixture (25-μL) contained one unit GoTaq^®^Hotstart polymerase (Promega, Madison, WI, USA), 0.6 μM of each primer, 2.5 mM MgCl2, 0.2 mM each dNTP, and 40 ng of template genomic DNA. Amplifications were performed in a DNA thermocycler (Applied Biosystems, Foster City, CA, USA) with the following temperature profile: an initial denaturation at 94°C for 3 min; 35 cycles each consisting of 30 s at 94°C, 30 s at 46°C, 30 s at 49°C, and 3 min at 72°C; and a final extension at 72°C for 10 min. The ERIC-PCR products were separated by electrophoresis on a 1.5% agarose gel with Goldview^®^staining (0.005%, v/v), and photographed using an UV Imaging System (GE Healthcare, Waukesha, WI, USA). The images were captured in TIFF file format for further analysis.

### Cluster Analysis

The observed bands in the gels were evaluated based on the presence (coded 1) or absence (coded 0) of polymorphic fragments for the ERIC and RAPD primers. Analysis of TIFF images was carried out using Gel Pro Analyzer (Version 4.0) and NTSYS-pc (Version 2.10), a numerical taxonomy and multivariate analysis software package (Rohlf, 2000). Similarity between fingerprints was determined by the Dice’s similarity coefficient at 1% band position tolerance and dendrograms were generated by unweighted pair group method using arithmetic average (UPGMA). The Simpson’s indexes of discrimination (DI) of ERIC-PCR and RAPD were calculated as described by Hunter and Gaston to determine the ability of each typing method (Hunter and Gaston, 1988).

### Random Amplified Polymorphic DNA

According to a previous study conducted by Chen et al. (2014), the 10-mer primer UBC155 (5′-CTGGCGGCTG-3′) was chosen for typing the *L. monocytogenes* isolates in this study (Farber and Addison, 1994). The PCR reaction condition used for the selected *L. monocytogenes* isolates and five reference strains was similar to the protocol described by Chen et al. (2014). The reaction mixtures were placed in a hot-lid cycler (Applied Biosystems, Foster City, CA, USA) and subjected to the following temperature profile: an initial five cycles at 94°C for 5 min, 35°C for 5 min, 72°C for 5 min; then 30 cycles each consisting of 1 min at 94°C, 2 min at 35°C, and 2 min at 72°C; and a final extension at 72°C for 10 min. The amplicons were electrophoresed on 1.5% agarose gel and photographed that were saved as TIFF file format for genotype analysis.

### Strain Library Construction

Strain library construction was performed as described in a previous study with minor modifications (Casarez et al., 2007). In brief, results of ERIC-PCR fingerprinting and multiplex PCR-based serogroups were used to screen isolates from the same sample in order to identify clonal isolates and assure as diverse a known source library as possible. Three to five *L. monocytogenes* isolates from each positive sample were fingerprinted by ERIC-PCR and serogroup analysis using multiplex PCR, and subsequently compared with each other. Isolates from the same sample with >90% similarity were considered as clonal. If the ERIC-PCR fingerprint of one isolate with >90% similarity from the same sample but has different serogroup determined by PCR, the isolate was included in the strain library. Only the isolate with >90% similarity ERIC-PCR fingerprint and the same serogroup from the same sample source were considered clonal. Clonal isolates from individual sample were excluded. At least one *L. monocytogenes* isolate from each known source sample was included in the library.

### Antimicrobial (AM) Susceptibility Test

Since no resistance criteria exist for *Listeria* antibacterial susceptibility test in Clinical and Laboratory Standards Institute guidelines for the tested AMs, criteria for *Staphylococcus aureus* were used except for where noted (**Table 1**; Clinical and Laboratory Standards Institute, 2014). A panel of 15 antimicrobials at the specific concentration per disk (Oxoid, Boston, MA, USA) was tested in this study (**Table 1**). Antimicrobial susceptibility test was performed as described in a previous study (Kovacevic et al., 2012). *S. aureus* ATCC 25923 and *Escherichia coli* ATCC 25922 were used as quality control strains for this study. Zones of inhibition were measured with a precision caliper to the nearest 0.01 mm. Isolates exhibiting resistance to three or more classes of antibiotics were considered as multidrug-resistant strains (Magiorakos et al., 2012).

**Table 1:** Antimicrobial resistance of foodborne *L. monocytogenes* isolates collected in South China.

Antimicrobial class	Antimicrobial agents (concentrations, μg or U)	Breakpoints (mm)			No. of isolates (%)		
		Susceptible	Intermediate	Resistant	Susceptible	Intermediate	Resistant
Aminoglycosides	Kanamycin (30)	≥18	14–17	≤13	167 (94.3)	7 (4.0)	3 (1.7)
	Gentamycin (10)	≥15	13–14	≤12	172 (97.2)	1 (0.6)	4 (2.2)
	Vancomycin (30)^∗^	≥17	15–16	≤14	172 (97.2)	5 (2.8)	0 (0)
Potentiated sulfonamide	Sulfamethoxazole with trimethoprim (23.75/1.25)	≥16	11–15	≤10	168 (94.4)	0 (0)	9 (5.6)
Tetracyclines	Doxycycline (30)	≥16	13–15	≤12	171 (96.6)	6 (3.4)	0 (0)
	Tetracycline (30)	≥19	15–18	≤14	164 (92.7)	7 (4.0)	6 (3.3)
Chloramphenicols	Chloramphenicol (30)	≥18	13–17	≤12	173 (97.8)	2 (1.1)	2 (1.1)
β-Lactam	Penicillin (10)	≥29	–	≤28	136 (76.8)	11 (6.2)	30 (16.9)
	Ampicillin (10)	≥29	–	≤28	140 (79.1)	7 (4.0)	30 (16.9)
	Sulbactam/ampicillin (10/10)	≥15	12–14	≤11	177 (100.0)	0 (0)	0 (0)
Fluoroquinolones	Levofloxacin (5)	≥19	16–18	≤15	158 (89.3)	17 (9.6)	2 (1.1)
	Ciprofloxacin (5)	≥21	16–20	≤15	134 (75.7)	40 (22.6)	3 (1.7)
Macrolides	Erythromycin (15)	≥23	14–22	≤13	174 (98.3)	2 (1.1)	1 (0.6)
Cephalosporins	Cephalothin (30)	≥18	15–17	≤14	172 (97.2)	5 (2.8)	0 (0)
Ansamycin	Rifampin (5)	≥20	17–19	≤16	172 (97.2)	1 (0.6)	4 (2.2)

*^∗^Breakpoint for *Entercococcus* spp.*

## Results

### Prevalence and Quantitative Analysis

**FIGURE 1:**
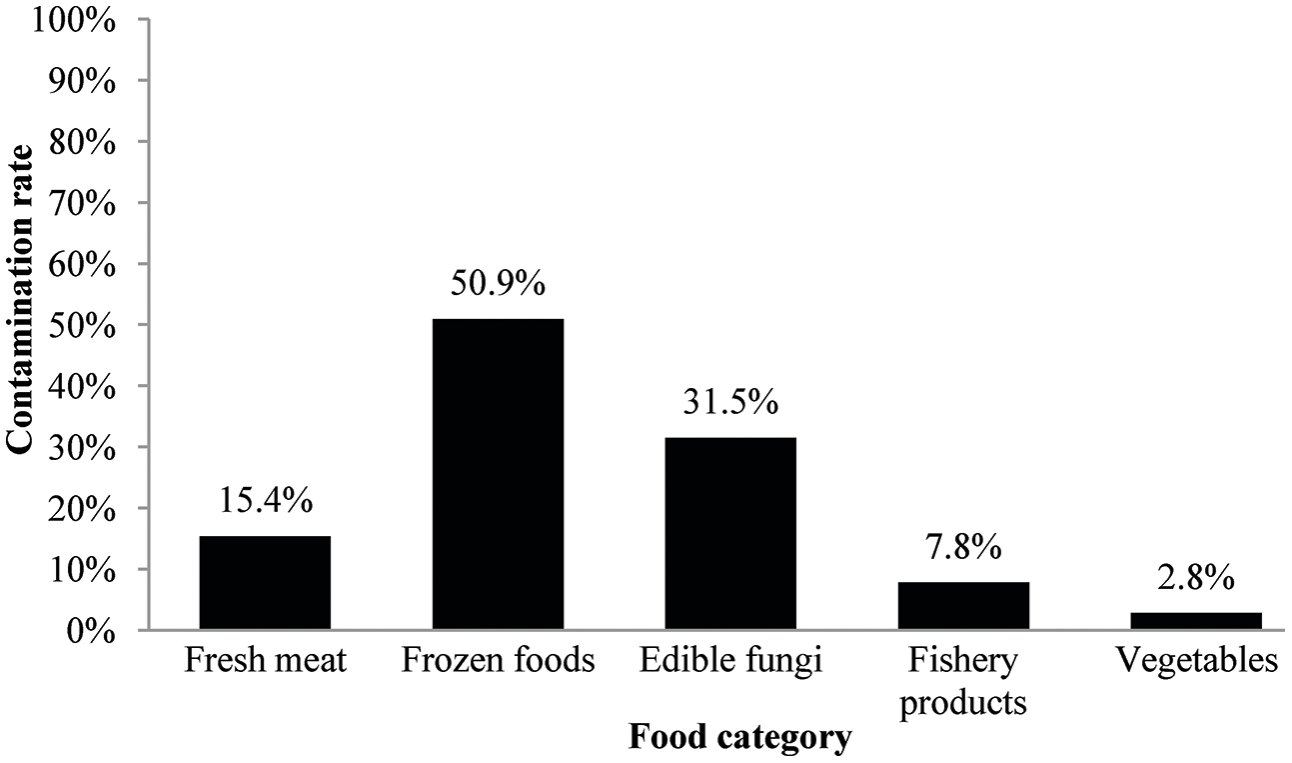
**Contamination rate of *Listeria monocytogenes* in different food products in South China**.

In this study, 123 (21.7%) samples were positive for *L. monocytogenes* out of 567 collected samples. Depending on the food category, the prevalence of *L. monocytogenes* in frozen food samples was 50.9% (56/110) and 31.5% in fresh edible fungus (34/108, including 29 *F. velutipes* samples and five other edible fungus samples), 15.4% in fresh meat (19/123), 7.8% in fishery products (12/154), and 2.8% in vegetables (2/72; **Figure 1**). Based on quantitative analysis, 75.0% (75/100) of the positive samples were contaminated at levels ranging between 0.3 and 10 MPN/g, that included 45 frozen foods, 12 fresh meat, 12 edible fungi, five fishery products, and one vegetable sample. Only in 14.0% of the positive samples the contamination level exceeded 100 MPN/g, which included 12 edible fungi samples, one frozen food, and one fishery product. The contamination level of *L. monocytogenes* in frozen food samples was low; in 95.8% (45/47) of the positive samples the levels were less than 10 MPN/g, only in one sample the levels exceeded 100 MPN/g. Surprisingly, 38.7% (12/31) of edible fungus samples were more than 100 MPN/g (**Table 2**).

**Table 2:** Distribution of *L. monocytogenes* in retail raw food samples collected from South China.

Food category	MPN value (MPN/g)		
	0.3 ≤ MPN < 10 (%)	10 ≤ MPN < 100 (%)	MPN ≥ 100 (%)
Fresh meat	12/14 (85.7)	2/14 (14.3)	0 (0)
Frozen foods	45/47 (95.8)	1/47 (2.1)	1/47 (2.1)
Edible fungi	12/31 (38.7)	7/31 (22.6)	12/31 (38.7)
Fishery products	5/7 (71.4)	1/7 (14.3)	1/7 (14.3)
Vegetables	1/1 (100.0)	0 (0)	0 (0)
**Total**	75/100 (75.0)	11/100 (11.0)	14/100 (14.0)

### Serogroup Analysis using Multiplex PCR

One hundred and seventy-seven *L. monocytogenes* isolates included in the strain library and five reference strains were used for serogroup analysis using multiplex PCR (Supplementary Table S2). As shown in **Table 3**, 42.4% (75/177) of the 177 *L. monocytogenes* isolates were recognized as serogroup I.1, of which isolates mainly isolated from fresh meat (13 isolates), frozen products (28 isolates), and edible fungi samples (31 isolates); 18.1% (32/177) as serogroup I.2, of which 28 isolates were recovered from frozen products samples; 7.3% as serogroup II.1, 26.0% (46/177) as serogroup II.2, which comprised of 20 isolates from edible fungi samples and 21 isolates from frozen products samples; and 6.2% as serogroup III. Additionally, serogroup I.1 (60.8%) and II.2 (39.2%) were identified in *L. monocytogenes* strains that were isolated from fresh edible fungi samples. Serogroup III (53.3%) was predominant in fishery product samples.

**Table 3:** Results of serogroup analysis carried out for the foodborne *L. monocytogenes* isolates collected in South China^#^.

Food category	Serogroup				
	I.1 (%)	I.2 (%)	II.1 (%)	II.2 (%)	III (%)
Fresh meat	13/26 (50.0)	3/26 (11.5)	7/26 (26.9)	1/26 (3.8)	2/26 (7.7)
Frozen foods	28/82 (34.1)	28/82 (34.1)	4/82 (4.9)	21/82 (25.6)	1/82 (1.2)
Edible fungi	31/51 (60.8)	0 (0)	0 (0)	20/51 (39.2)	0 (0)
Fishery products	3/15 (20.0)	1/15 (6.7)	1/15 (6.7)	2/15 (13.3)	8/15 (53.3)
Vegetables	0 (0)	0 (0)	1/3 (66.7)	2/3 (33.3)	0 (0)
**Total**	75/177 (42.4)	32/177 (18.1)	13/177 (7.3)	46/177 (26.0)	11/177 (6.2)

*#The 177 *L. monocytogenes* isolates included in the strain library were used for statistical analysis.*

### ERIC-PCR Typing

**FIGURE 2:**
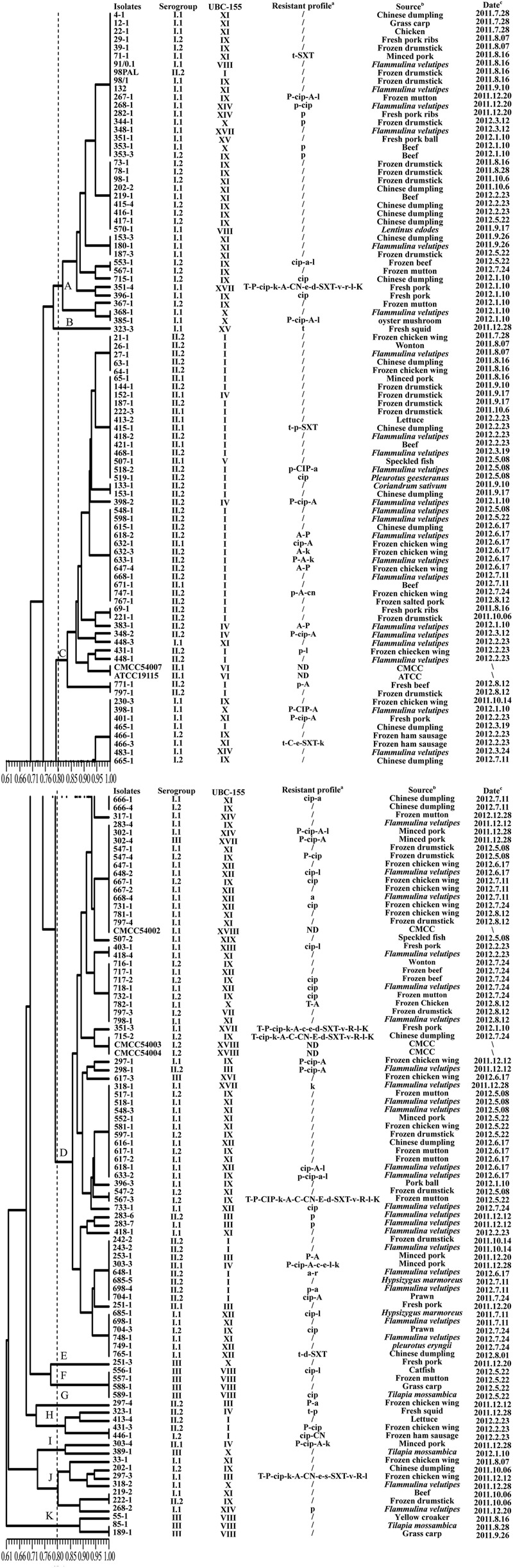
**Characterization of foodborne L. monocytogenes isolates obtained in food products from South China.** The dendrogram was constructed based on ERIC-PCR analysis; a: T (t), tetracycline; P (p), penicillin; CIP (cip), ciprofloxacin; K (k), kanamycin, A (a), ampicillin; C (c), cephalothin; CN (cn), gentamycin, E (e), erythromycin, D (d), doxycycline; SXT (sxt), sulfamethoxazole with trimethoprim; V (v): vancomycin; R (r), rifampin; L (l), levofloxacin; /, no resistance; ND, not detected; Name of antibiotics with capital letters implies resistance; Name of antibiotics with lowercase letters implies intermediate resistance. b: -, no gene absent; genes listed mean absent. c: CMCC, China Medical Culture Collection; ATCC, American Type Culture Collection. d: ∖, unknown.

Using ERIC-PCR, genomic DNA of *L. monocytogenes* isolates displayed DNA bands in sizes of between 200 and 2200 bp, most of isolates from the same sample generated similar fingerprints. Sixty ERIC-types (DI = 0.960) were classified among 177 foodborne isolates. Based on cluster analysis of genetic profiles obtained from ERIC-PCR typing (**Figure 2**), there were three singletons and eight clusters at a relative similarity coefficient of 80%; isolates belonging to a distinct serogroup clustered together, such as in cluster D that included the strains 283-4, 298-1, 302-4, 547-4, and 648-1. It is noteworthy that 23 isolates, from *F. velutipes* samples, belonging to serogroup I.1 fell into cluster D, which included strains 298-1, 318-1, 518-1, 548-3, 618-1, 633-2, 733-1, 283-4, 648-2, 668-4, 398-1, 483-1, 418-4, 718-1, 798-1, 283-6, 283-7, 418-1, 243-2, 648-1, 698-4, 698-1, and 748-1. For fishery products, four isolates (556-1, 557-1, 588-1, and 589-1) shared the same ERIC-type under cluster F; three isolates (55-1, 85-1, and 189-1) shared similar genetic fingerprints as in cluster K, and all these seven isolates belonged to serogroup III.

### RAPD Genotyping Analysis

**FIGURE 3:**
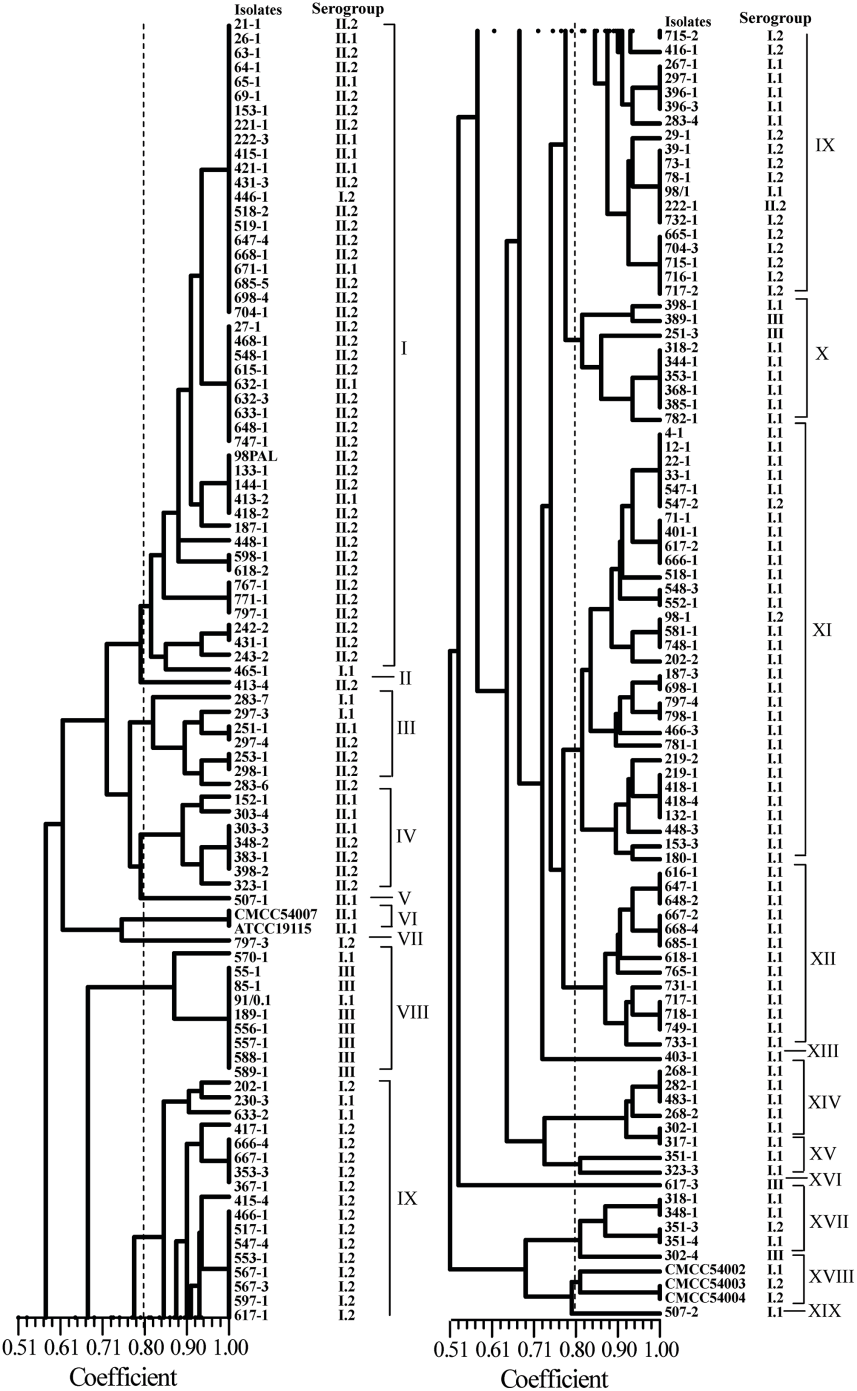
**Random amplified polymorphic DNA (RAPD) analyses of foodborne *L. monocytogenes* isolated from food products in South China**.

Using UBC-155 primer, it was determined that at a relative similarity coefficient of 80%, 177 isolates and five reference strains fell into 14 clusters and five singletons (designated as I to XIX; **Figure 3**). The discriminatory index of RAPD typing for the 177 isolates was 0.972 based on Simpson’s Index of diversity (Hunter and Gaston, 1988). The strong correlation between RAPD-type and serogroup was observed using RAPD typing (**Figure 3**), cluster I included serogroup II.2 and II.1, which accounted for 78.3% (36/46) and 17.4% (8/46), respectively. Most of the isolates in cluster VIII belonged to serogroup III (87.5%); serogroup I.2 (27/36) dominated in cluster IX and serogroup I.1 dominated in cluster X (77.8%), XI (93.1%), and XII (100%). As shown in **Figure 2**, when comparing the isolates included in the clusters of ERIC-PCR and RAPD, an agreement was observed with ERIC-PCR and RAPD subtyping, i.e., isolates in cluster XI and IX typed using RAPD belonged to cluster I of ERIC-PCR).

### Antimicrobial Susceptibility Test

Antimicrobial susceptibility of 177 *L. monocytogenes* isolates were evaluated using the disk diffusion method. Based on the breakpoint criteria for *S. aureus* or *Enterococcus* spp., 42 antimicrobial susceptibility profiles were identified in this study (**Figure 2**). Penicillin (23.1%), ampicillin (20.9%), and ciprofloxacin (24.3%) were the three most frequent antimicrobial resistance profiles among the *L. monocytogenes* isolates, while the resistance rate of tetracycline and doxycycline was 7.3 and 3.4%, respectively. For the other 12 antibacterial agents, all 177 isolates of *L. monocytogenes* displayed 89.3–100% susceptibility (**Table 1**). Considering the fact that an intermediate resistant isolate may become a resistant strain under certain circumstances (Ruiz-Bolivar et al., 2011), all 177 isolates were grouped into 42 antibacterial resistance patterns, including 106 (59.9%) isolates of *L. monocytogenes* that were susceptible to the 15 tested antibacterial agents and 10 (5.6%) isolates that were multidrug resistant (**Figure 2**). Notably, 83.3% of the isolates (10/12) that were resistant to up to four antimicrobials were isolated from the livestock and poultry meat and were associated food products (**Figure 2**).

## Discussion

In this study, we collected 567 food samples including raw meat, frozen foods, edible fungi, fishery products, and vegetables from16 cities or districts in South China. The contamination rate of *L. monocytogenes* (21.7%) in food samples from South China was in consistent with that in Southeastern China as shown by Wang et al. (2013). Over 15% of fresh meat samples were positive for *L. monocytogenes* in the present study, however, raw meat and poultry are not considered at high risk for causing foodborne listeriosis provided adequate cooking precedes consumption and that cross-contamination is avoided. Nevertheless, raw or insufficiently cooked meats may serve as sources of cross-contamination for products that are intended to be consumed without heat treatment, and along with inadequate cleaning and sanitation, these foods have been recognized as the main sources of post-processing contamination of RTE meat products (Lianou and Sofos, 2007; Sauders et al., 2009). Quantitative analysis revealed that over 75% of *L. monocytogenes*-positive samples had counts below 10 MPN/g, which was consistent with the results of the retail raw foods collected in Northeast of China (Wang et al., 2013). In the present study, the contamination rate of frozen foods was up to 50.9%, while the MPN value of positive samples were mostly below 10 MPN/g, these results may associated with the tolerance ability of *L. monocytogenes* in low temperature environments (Vazquez-Boland et al., 2001). Surprisingly, 76.3% (29/38) of *F. velutipes* samples were positive for *L. monocytogenes* and the MPN value of 12 positive samples exceeded 100 MPN/g, while the contamination rates of other edible fungi samples were low (data not shown), these data were significantly higher than that reported in other countries as reviewed by Lianou and Sofos (2007). To date, limited data have been available about the prevalence of *L. monocytogenes* in edible fungi products in China. In addition, there are no specific qualitative and quantitative standards to determine the presence of *L. monocytogenes* in the mushroom products. However, some countries have formulated a zero-tolerance policy for *L. monocytogenes* in mushroom products, such as in the United States and Canada (Canadian Food Inspection Agency, 2012; U. S. Food and Drug Administration, 2012). It is necessary to draft a corresponding microbiological standard to ensure the quality of mushroom products in China.

Previous studies reported over 95% of the isolates in human listeriosis and food samples belonged to serotype 1/2a, 1/2b, 1/2c, and 4b (Pontello et al., 2012; Althaus et al., 2014). In this study, 42.4% of *L. monocytogenes* isolates belonged to serogroup I.1 (serotype 1/2a-3a), followed by serogroup II.2 (26.0%), serogroup I.2 (18.1%), serogroup II.1 (7.3%), and serogroup III (6.2%). Serogroup I.1 (serotype 1/2a-3a) predominant among the foodborne isolates, the serogroup compositions of these foodborne isolates was in agreement with that previously reported for other foodborne isolates (Chen et al., 2009; Korsak et al., 2012). Interestingly, isolates recovered from edible fungi samples only belonged to serogroup I.1 and II.2, while Viswanath et al. (2013) reported only serotype 4a contamination in a small-scale mushroom production facility. Serogroup III was rarely isolated from foods (Orsi et al., 2011), however, in this study 11 isolates belonging to serogroup III were isolated from food samples; 72.7% (8/11) isolates were from fishery products, Korsak et al. (2012) reported that serogroup III was mainly isolated from vegetables in Poland. These results indicated that some specific serogroups of *L. monocytogenes* may have distinct ecological niches; Ochiai et al. (2010) also reported this for *L. monocytogenes* isolated from retail meat in Japan. Continuous and comprehensive investigation should be carried out for better understanding of the ecology of *L. monocytogenes*.

As contaminated foods are considered as the transmission source for human clinical listeriosis, we decided to carry out our investigation using *L. monocytogenes* strains that were isolated from food samples in South China, and examining the diversity of their antimicrobial profiles. In the present study, all of the *L. monocytogenes* isolates were susceptible to sulbactam/ampicillin (**Table 1**) and over 90% of isolates were susceptible to 10 antibacterial agents, namely kanamycin, gentamycin, sulfamethoxazole with trimethoprim, doxycycline, chloramphenicol, erythromycin, cephalothin, rifampin, tetracycline, and vancomycin, which indicated that these antibacterial agents are still effective for the treatment of listeriosis. The tetracycline and ciprofloxacin resistance rates were significantly different than that of the *L. monocytogenes* isolated in Northern China as described by Yan et al. (2010), which is possibly due to the influence of geographic differences. Surprisingly, 20% of *L. monocytogenes* isolates exhibited resistance toward the first-choice drug penicillin and ampicillin (**Table 1**), although no penicillin-resistant strain was found in the previous studies conducted in China (Chao et al., 2007; Yang et al., 2008; Zhao et al., 2012). Therefore, more attention is required while monitoring the variation in the trend of resistance toward penicillin and ampicillin. These findings indicated that *L. monocytogenes* is slowly becoming resistant to antibiotics in South China. This emphasized the need to continuously conduct surveillance for antibacterial susceptibility of foodborne *L. monocytogenes* in China.

In the present study, 5.6% (10/177) of *L. monocytogenes* isolates were determined as multidrug resistant (**Figure 2**) and ten isolates that were resistant to four antibiotics were obtained from animal foods and its associated products. These results support that the use of antibiotics in poultry and livestock as growth promoter has led to the emergence of antimicrobial-resistant bacteria in the food and associated environment (Harakeh et al., 2009; Wieczorek et al., 2012). These results also indicated that foods from both poultry and livestock are possibly the reservoir of multidrug resistant *L. monocytogenes* strains. However, the results of multidrug resistance were inconsistent with that previously reported (Yan et al., 2010; Zhao et al., 2012), which can be attributed to the different source-composition of foodborne *L. monocytogenes* as well as different criteria used to determine multidrug resistance. In addition, it should be noted that, in our study 31.4% (16/51) isolates recovered from edible fungi samples exhibited resistance to more than two antibiotics. To the best of our knowledge, the processes involved in the production of edible fungi in scale-level plants would not use any antibiotics to prevent edible fungi contaminating other harmful microbes. This is the first report that *L. monocytogenes* isolates recovered from edible fungi samples demonstrated a high frequency of multidrug resistance, indicating that mushrooms may serve as the potential reservoirs of multidrug-resistant strains of *L. monocytogenes*.

Recently, high discriminatory molecular typing methods, i.e., PFGE, RAPD, and ERIC-PCR, have been developed for the differentiation of pathogenic bacteria. ERIC-PCR and RAPD techniques have been extensively used to determine genetic lineages of *L. monocytogenes* (Zhou and Jiao, 2004; Aurora et al., 2009; Chen et al., 2010). In the present study, ERIC-PCR and RAPD also yielded comparable results for the typing of *L. monocytogenes* strains, the discriminatory ability of ERIC-PCR and RAPD were 0.960 and 0.972, respectively. Genetic relatedness analysis revealed that there were no prominent associations between specific food types, antibiotic resistance, serogroups, and genetic diversity. The clustering results generated were different depending on the typing methods used (**Figures 2** and **3**), because the typing methods possibly target different genetic markers (Marshall et al., 1999). Seven isolates (418-1, 418-4, 518-1, 548-3, 698-1, 748-1, and 798-1) belonging to serogroup I.1 were grouped into ERIC-D and RAPD-XI; ten isolates (27-1, 398-2, 418-2, 468-1, 518-2, 548-1, 598-1, 618-1, 633-1, 668-1) belonging to serogroup II.2 were clustered into ERIC-C and RAPD-I. These isolates were obtained from *F. velutipes* samples, indicating that these isolates share similar genetic information and can better tolerate the disinfectants and sanitizers used in the mushroom production facility. Further studies are likely to elucidate the characteristics of these specific subtypes of *L. monocytogenes* colonizing the processing environments that in turn will aid in exploring better cleaning and sanitation measures. Previous studies demonstrated that the antibacterial resistance in *L. monocytogenes* was possibly acquired *via* self-transferable plasmids (Poyart-Salmeron et al., 1990), conjugative mobilization (Charpentier et al., 1999; Toomey et al., 2009), and eﬄux pumps (Rakic-Martinez et al., 2011; Jiang et al., 2012). Interestingly, two multidrug-resistant isolates (351-3, 715-2) were clustered together as revealed by ERIC-PCR, while isolates 351-3, 351-4 recovered from the fresh pork samples displayed unique fingerprints based on RAPD typing, suggesting that the multidrug-resistant isolates may acquire resistance by vertical and horizontal gene transfer. Further studies are required to elucidate the underlying molecular mechanisms for the acquisition of antimicrobial resistance by *L. monocytogenes*.

## Conclusion

Overall, the findings of this study demonstrated high contamination rate of *L. monocytogenes* in raw foods in South China, while the MPN values were relative low. Serogroup I.1 and II.2 were dominant among the foodborne *L. monocytogenes* strains, indicating that some specific serogroups of *L. monocytogenes* may have distinct ecological niches. Approximately 59.9% of the strains were susceptible to 15 antibiotics. All 177 isolates were grouped into 42 antibacterial susceptibility profiles and 5.6% of the isolates were multidrug resistant. Both ERIC-PCR and RAPD displayed excellent discriminatory ability for typing *L. monocytogenes*, the discriminatory index of ERIC-PCR and RAPD were 0.960 and 0.972, respectively. The present study provided the first baseline data on the prevalence, contamination level, and characteristics of *L. monocytogenes* isolated from raw foods in South China. Some multidrug resistant strains belonged to an epidemiologically important serogroup (I.1 and II.1), implying a potential public health risk. In addition, Chinese food safety authorities should draft appropriate standards to control *L. monocytogenes* contamination for the improvement of microbiological safety of retail raw foods.

## Conflict of Interest Statement

The authors declare that the research was conducted in the absence of any commercial or financial relationships that could be construed as a potential conflict of interest.
