# Supplementary material for: Prevalence, enumeration, and pheno- and genotypic characteristics of Listeria monocytogenes isolated from raw foods in South China
Source: Front Microbiol. 2015 Sep 29;6:1026. doi: 10.3389/fmicb.2015.01026 (PMC4586447; doi:10.3389/fmicb.2015.01026)
Supplement: Supplementary file 1 [file Table_1.DOCX]

Table S1 Primers used for identification of the serogroup and virulence genes of *L. monocytogenes* strains

| PCR tests | Target genes | Forward and reverse primers (5'–3') | Specificity | Annealing temperature (°C) | Size of PCR amplicon (bp) | Reference |
| --- | --- | --- | --- | --- | --- | --- |
| Serovar typing | Lmo0737 | AGGGCTTCAAGGACTTACCC | *L. monocytogenes* serovars 1/2a, 3a, 1/2c and 3c | 53 | 691 | Doumith, et al., 2004 |
|  |  | ACGATTTCTGCTTGCCATTC |  |  |  |  |
|  | Lmo1118 | AGGGGTCTTAAATCCTGGAA | *L. monocytogenes* serovars 1/2c and 3c | 53 | 906 |  |
|  |  | CGGCTTGTTCGGCATACTTA |  |  |  |  |
|  | ORF2819 | AGCAAAATGCCAAAACTCGT | *L. monocytogenes* serovars 1/2b, 3b, 4b, 4d, 4e and 7 | 53 | 471 |  |
|  |  | CATCACTAAAGCCTCCCATTG |  |  |  |  |
|  | ORF2110 | AGTGGACAATTGATTGGTGAA | *L. monocytogenes* serovars 4b, 4d and 4e | 53 | 597 |  |
|  |  | CATCCATCCCTTACTTTGGAC |  |  |  |  |
|  | prs | GCTGAAGAGATTGCGAAAGAAG | All *listeria* species | 53 | 370 |  |
|  |  | CAAAGAAACCTTGGATTTGCGG |  |  |  |  |
