# Supplementary material for: Prevalence, enumeration, and pheno- and genotypic characteristics of Listeria monocytogenes isolated from raw foods in South China
Source: Front Microbiol. 2015 Sep 29;6:1026. doi: 10.3389/fmicb.2015.01026 (PMC4586447; doi:10.3389/fmicb.2015.01026)
Supplement: Supplementary file 2 [file Table_2.DOCX]

Table S2 The isolates in the strains library screened by the combination of serogroup and ERIC-PCR analysis

| **No.** | isolates | **No.** | isolates | **No.** | isolates | **No.** | isolates | **No.** | isolates | **No.** | isolates |
| --- | --- | --- | --- | --- | --- | --- | --- | --- | --- | --- | --- |
| **1** | CMCC54002 | **31** | 152-1 | **61** | 298-1 | **91** | 413-4 | **121** | 552-1 | **151** | 667-1 |
| **2** | CMCC54003 | **32** | 153-1 | **62** | 302-1 | **92** | 415-1 | **122** | 553-1 | **152** | 667-2 |
| **3** | CMCC54004 | **33** | 153-3 | **63** | 302-4 | **93** | 415-4 | **123** | 556-1 | **153** | 668-1 |
| **4** | CMCC54007 | **34** | 180-1 | **64** | 303-3 | **94** | 416-1 | **124** | 557-1 | **154** | 668-4 |
| **5** | ATCC19115 | **35** | 187-1 | **65** | 303-4 | **95** | 417-1 | **125** | 567-1 | **155** | 671-1 |
| **6** | 4-1 | **36** | 187-3 | **66** | 317-1 | **96** | 418-1 | **126** | 567-3 | **156** | 685-1 |
| **7** | 12-1 | **37** | 189-1 | **67** | 318-1 | **97** | 418-2 | **127** | 570-1 | **157** | 685-5 |
| **8** | 21-1 | **38** | 202-1 | **68** | 318-2 | **98** | 418-4 | **128** | 581-1 | **158** | 698-1 |
| **9** | 22-1 | **39** | 202-2 | **69** | 323-1 | **99** | 421-1 | **129** | 588-1 | **159** | 698-4 |
| **10** | 26-1 | **40** | 219-1 | **70** | 323-3 | **100** | 431-1 | **130** | 589-1 | **160** | 704-1 |
| **11** | 27-1 | **41** | 219-2 | **71** | 344-1 | **101** | 431-3 | **131** | 597-1 | **161** | 704-3 |
| **12** | 29-1 | **42** | 221-1 | **72** | 348-1 | **102** | 446-1 | **132** | 598-1 | **162** | 715-1 |
| **13** | 33-1 | **43** | 222-1 | **73** | 348-2 | **103** | 448-1 | **133** | 615-1 | **163** | 715-2 |
| **14** | 41-1 | **44** | 222-3 | **74** | 351-1 | **104** | 448-3 | **134** | 616-1 | **164** | 716-1 |
| **15** | 55-1 | **45** | 230-3 | **75** | 351-3 | **105** | 465-1 | **135** | 617-1 | **165** | 717-1 |
| **16** | 63-1 | **46** | 242-2 | **76** | 351-4 | **106** | 466-1 | **136** | 617-2 | **166** | 717-2 |
| **17** | 64-1 | **47** | 243-2 | **77** | 353-1 | **107** | 466-3 | **137** | 617-3 | **167** | 718-1 |
| **18** | 65-1 | **48** | 251-1 | **78** | 353-3 | **108** | 468-1 | **138** | 618-1 | **168** | 731-1 |
| **19** | 69-1 | **49** | 251-3 | **79** | 367-1 | **109** | 483-1 | **139** | 618-2 | **169** | 732-1 |
| **20** | 71-1 | **50** | 253-1 | **80** | 368-1 | **110** | 507-1 | **140** | 632-1 | **170** | 733-1 |
| **21** | 73-1 | **51** | 267-1 | **81** | 383-1 | **111** | 507-2 | **141** | 632-3 | **171** | 747-1 |
| **22** | 78-1 | **52** | 268-1 | **82** | 385-1 | **112** | 517-1 | **142** | 633-1 | **172** | 748-1 |
| **23** | 85-1 | **53** | 268-2 | **83** | 389-1 | **113** | 518-1 | **143** | 633-2 | **173** | 749-1 |
| **24** | 91/0.1 | **54** | 282-1 | **84** | 396-1 | **114** | 518-2 | **144** | 647-1 | **174** | 765-1 |
| **25** | 98/1 | **55** | 283-4 | **85** | 396-3 | **115** | 519-1 | **145** | 647-4 | **175** | 767-1 |
| **26** | 98PAL | **56** | 283-6 | **86** | 398-1 | **116** | 547-1 | **146** | 648-1 | **176** | 771-1 |
| **27** | 98-1 | **57** | 283-7 | **87** | 398-2 | **117** | 547-2 | **147** | 648-2 | **177** | 781-1 |
| **28** | 132-1 | **58** | 297-1 | **88** | 401-1 | **118** | 547-4 | **148** | 665-1 | **178** | 782-1 |
| **29** | 133-1 | **59** | 297-3 | **89** | 403-1 | **119** | 548-1 | **149** | 666-1 | **1791** | 797-1 |
| **30** | 144-1 | **60** | 297-4 | **90** | 413-2 | **120** | 548-3 | **150** | 666-4 | **180** | 797-3 |
| **181** | 797-4 | **182** | 798-1 |  |  |  |  |  |  |  |  |
